# Conventional or Interpersonal Communication: Which Works Best in Disseminating Malaria Information in an Endemic Rural Bangladeshi Community?

**DOI:** 10.1371/journal.pone.0090711

**Published:** 2014-03-06

**Authors:** Syed Masud Ahmed, Mohammad Shamim Hossain, Moktadir Kabir

**Affiliations:** 1 Centre for Equity and Health Systems, icddr,b, Dhaka, Bangladesh; 2 James P. Grant School of Public Health, BRAC Institute of Global Health (BIGH), BRAC University, Dhaka, Bangladesh; 3 Centre of Excellence for Universal Health Coverage, James P. Grant School of Public Health, BRAC Institute of Global Health (BIGH), BRAC University, Dhaka, Bangladesh; 4 Research and Evaluation Division, BRAC, Dhaka, Bangladesh; 5 Health, Nutrition and Population Programme, BRAC, Dhaka, Bangladesh; Johns Hopkins University, United States of America

## Abstract

**Background:**

Since 2007, BRAC has been implementing malaria prevention and control programme in 13 endemic districts of Bangladesh under the National Malaria Control Programme. This study was done to examine the role of different communication media in bringing about changes in knowledge and awareness which facilitate informed decision-making for managing malaria-like illnesses.

**Methods:**

A baseline survey in 2007 before inception of the programme, and a follow-up survey in 2012 were done to study changes in different aspects of programme interventions including the communication component. Both the surveys used the same sampling technique to select 25 households at random from each of the 30 mauza/villages in a district. A pre-tested, semi-structured questionnaire was used to collect relevant information from respondents in face-to-face interview. Analysis was done comparing the study areas at two different times. Statistical tests were done as necessary to examine the differences.

**Results:**

The intervention succeeded in improving knowledge in some trivial areas (e.g., most frequent symptom suggestive of malaria, importance of using insecticidal bed nets) but not in critical domains necessary for taking informed action (e.g., mode of malaria transmission, awareness about facilities providing free malaria treatment). Inequity in knowledge and practice was quite common depending upon household affluence, location of households in high or low endemic districts, and sex. Of the different media used in Information, Education and communication (IEC) campaigns during the study period, interpersonal communication with community health workers/relatives/neighbours/friends was found to be more effective in improving knowledge and practice than conventional print and audio-visual media.

**Conclusion:**

This study reiterates the fact that conventional media may not be user-friendly or culture-sensitive for this semi-literate/illiterate community where dissemination through ‘words of mouth’ is more common, and as such, interpersonal communication is more effective. This is especially important for initiating informed action by the community in managing malaria-like illnesses.

## Introduction

Communication for health is essential for building knowledge and awareness, reducing stigma and other barriers and promote behavior change, motivating community for uptake of already available interventions, and taking informed action for prevention and treatment [1]. Effective communication plays a key role in adherence to particular intervention or intervention component [2]. It has been seen that communication becomes most effective when it is integrated with other programme activities and when consistent messages are conveyed through a mix of channels round the year [3]–[5]. Among the different channels of communication, interpersonal communication has been shown to be more effective in situations of poor literacy and general low level of health awareness [6], [7]. The interaction between and communication among community members facilitate the ‘use of untapped resource of social capital’ [8] and promote essential communication for malaria control [9]. This is because Information, Education and Communication (IEC) is a ‘process of working with individuals and communities to develop communication materials/tools to promote positive behaviors which are appropriate to their settings’ [10].

**Figure 1:**
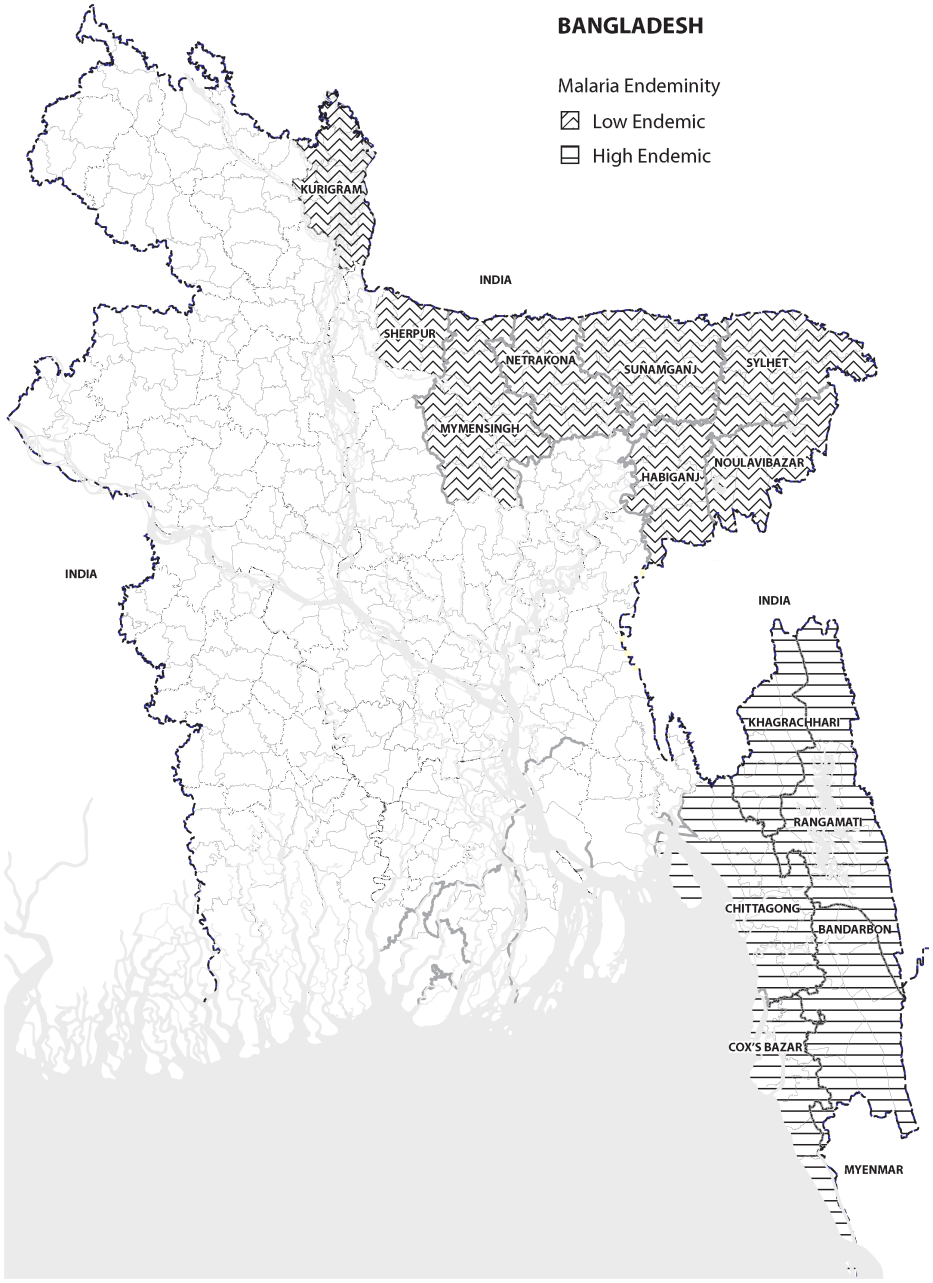
Malaria endemic districts of Bangladesh.

About 98% malaria mortality and morbidity in Bangladesh is reported from 13 malaria endemic districts bordering India and Myanmar [11], overall prevalence being 3.1% according to Rapid Diagnostic Test) [12]. These malaria endemic districts is divided into five high endemic south-eastern districts (Chittagong, Cox's Bazar, Khagrachari, Rangamati, Bandarban; prevalence 7.2%) and eight low endemic north-eastern districts (Kurigram, Sherpur, Mymensingh, Netrokona, Sunamgonj, Sylhet, Moulvibazar, Habiganj; prevalence 0.5%) (Fig. 1) [12]. Of the former, the three Chittagong Hill Tract (CHT) districts (Khagrachari, Rangamati, Bandarban) bordering India and Myanmar have the highest prevalence (11%). BRAC, an indigenous non-governmental development organization (NGO) in Bangladesh, had been implementing malaria prevention and control programme in 13 malaria-endemic districts under the National Malaria Control Programme since 2007. This is a collaborative effort of the government of Bangladesh and a BRAC-led NGO consortium consisting of 20 smaller partner NGOs [13]. The overall goal of the programme was to reduce the burden of malaria in the endemic areas by 2012.

BRAC's malaria control and prevention programme has both preventive (insecticidal bed net distribution and IEC activities) and curative (presumptive case management, early diagnosis and prompt treatment, and referral of complicated cases to higher facilities) components [13]. Various IEC materials such as posters, leaflets, billboards, radio and television spots, etc. are used for dissemination of relevant information and building awareness for affirmative action. The community health workers use flip charts in interpersonal communication with women and other household members during home visits. Besides, other means such as folk songs, popular theatre, loudspeaker miking, etc. are used sparingly to mobilize the community for informed action regarding malaria control and prevention [13].

A baseline survey in 2007 before inception of the programme [14], and a follow-up survey in 2012 [15] was done to study changes in different aspects of the programme interventions such as key knowledge and practices related to the prevention and treatment of malaria, including the IEC component. This paper reports on these changes, and examines the role of different communication medias during 2007-'12 in bringing about these changes.

## Materials and Methods

The baseline survey (Jul.-Nov. 2007) was conducted in all the 13 malaria endemic districts while the follow-up survey (Jan.–Feb. 2012) was conducted in eight endemic districts (five high endemic districts and three low endemic districts with highest prevalence of malaria e.g., Habiganj (1.74%), Sunamganj (1.07%), and Mymensingh (0.4%)) [12]. Beside information on socioeconomic and demographic characteristics and knowledge and practice regarding prevention and management of malaria, information on access to and use of different communication channels such as print media, audiovisual media, interpersonal communication, and other forms of dissemination such as loud-speaker campaigns in gatherings in rural bazaars or fairs etc. was also collected.

### Ethical considerations

The study passed through the institutional review process at BRAC Research and Evaluation Division (RED) for ethical approval. ‘The RED Coordination Committee’ (comprised of senior lead researchers) reviewed the research protocol including its ethical aspects and method of consent taking and approved it for field implementation. The study did not have any invasive procedure and information was collected through face-to-face interview. Since the respondents comprised largely of an illiterate/semiliterate community, and since the rural community in Bangladesh is largely skeptical of signing any written documents for historical reasons, written consent was not feasible. Instead, we opted for informed verbal consent from the respondents before conducting the interview. This is the main form of consent taken in conducting non-invasive studies such as questionnaire surveys in Bangladesh. In this process, the written consent was read to the respondent with necessary explanation in the presence of a literate witness. When the interviewer was satisfied that the respondent understood it including its implications, and had agreed to participate, only then was s/he included in the survey and interviewed. For record, the written consent was preserved with ID of the respondent with a note that verbal consent was taken in the presence of a witness. Anonymity of the respondents was maintained at all stages of data analysis. Data were used for research purpose only.

### Sample design and sample size

The same sampling design was adopted in both the surveys. The primary sampling unit was mauza/village. From each of the selected districts, 30 Mauzas/villages were selected using a probability proportional to size (PPS) sampling procedure. Twenty-five households were selected from each mauza/village by systematic random sampling method. Sample size was calculated using web-based software (C-Survey 2.0) based on the conservative estimates of malaria prevalence of 2%, design effect of 2, and precision of 1.5% at 95% confidence interval. Thus, 750 households were included from each district. One respondent from each household was interviewed.

### Tools development

A structured questionnaire was developed incorporating all aspects of the objectives of the survey(s) and was pre-tested in a village outside our sample for ascertaining consistency, appropriateness of languages, sequencing of the questions, and to have an insight into the field operation procedure. The questionnaire was finalized incorporating relevant feedbacks received from field testing.

### Recruitment, training and deployment of the interviewers

The survey team comprised of experienced interviewers (social science graduates/anthropologists with survey experiences) and their supervisors. In hilly areas, interviewers from different ethnicity were recruited to interview respective ethnic groups of people. A five-day intensive training was given to the interviewers consisting of didactic lectures, mock interviews, role play and field practice at community level. Several teams were working in paralell in each district. Each team typically consisted of 3–4 members including one supervisor.

### Field operation

One or two days before the beginning of the survey, the teams were deployed in study mouzas/villages for rapport building. During this time, the villagers were informed about the purpose and activities of the survey and seek their cooperation. In each village, the study team drew a field map. Households were then chosen through a systematic random sampling process e.g., every nth household was selected as the team moved from the center or periphery of the mauza following a designated path using the “spin the bottle” method [16]. Households were visited on three repeated occasions at intervals, if the first attempt was not successful due to absence of the respondents. When these repeated attempts failed, the interview was called-off for that particular household. The questionnaires were administered to the household head or spouse (or any knowledgeable member in their absence) in face-to-face interview using a pre-tested structured questionnaire.

The day-to-day field activities of the teams were fine-tuned by field researchers based in local offices. The investigators from central office at Dhaka made frequent field visits for checking the quality of interviews and providing assistance and guidance. Whenever necessary, re-interview was done by the supervisors for securing reliable and valid data.

### Analysis of data

Analysis was done comparing the two endemic areas at two different time such as baseline in 2007 and follow-up in 2012. Statistical tests were done as necessary (t-test, χ^2^) to examine differences. Only the selected variables were compared to explore changes in key areas of the programme. While running logistic regression, all the print and audiovisual medias were merged into a single category ‘mass media’ and all interpersonal communications between respondent and the CHWs, neighbours/relatives/friends were merged into a single category ‘interpersonal communication’. The ‘other’ category included dissemination of information with loud speakers (miking) at gathering of people e.g., in fairs, weekly bazaars, etc.

The assets included for developing the wealth index were: table, bed-cot, quilt, watch, radio, television, bi-cycle and electricity. Each of the variables was recorded into categorical dichotomous (yes, no) variable. Eight dichotomous variables were created and standardized. The principal component analysis was run with all constructed variables with certain criteria. The component score coefficient matrix was multiplied by the standardized variables to produce factor scores which were termed as household wealth score. The wealth scores were further classified into five quintiles, starting from the lowest (1st quintile, poorest) to highest (5th quintile, least poor).

## Results

Table 1 shows some key knowledge indicators comparing baseline (2007) and the follow-up survey (2012). There were improvements in some of the knowledge indicators, sometime dramatic as in the case of preventive property of insecticidal bed nets (from 1–2% to 70–80%), but decline was also observed for some critical indicators such as mode of transmission (from 33–40% to around 20%) and place where malaria treatment can be accessed free of cost (from 60–70% to around 53%). Proportion of households with at least one insecticidal bed net increased from 2.2% in 2007 to 76% in 2012, but not knowledge on the norms of using it (data not shown).

**Table 1:** Key malaria knowledge indicators in 2007 (baseline) and 2012 (follow-up) by areas %.

	High endemic districts			Low endemic districts			
			χ^2^ value			χ^2^ lue	All
*Knowledge on malaria*	2007	2012	(95% CI)	2007	2012	(95% CI)	
Knows that malaria is caused by bite of	93.4	95.3	12.05	95.3	96.4	4.70	95.7
mosquito			(11.8–12.80			(1.9–11.9)	
							
Knows that malaria is transmitted by the bite	40.0	24.2	204.48	32.8	19.7	647.90	22.5
of mosquito which has bitten a malaria			(206.3–210.70			(639.6–656.3)	
patient							
							
Knows the most common symptom of	78.3	95.4	471.29	83.9	92.5	101.30	
Malaria*			(465.5–477.1)			(100.4–102.2)	94.3
							
Knows that insecticidal bed nets prevent	1.0	70.5	3753.34	2.2	81.0	5618.61	
malaria			(3692.2–3815.5)			(5522.6–5716.3)	74.4
							
Knows that malaria is treated by allopathic	98.7	99.3	6.89	99.3	99.5	0.87	
medicine			(5.0–9.6)			(0.3–2.4)	99.3
							
Knows that malaria treatment is available in	60.1	54.1	26.81	72.4	52.9	281.07	53.7
public and NGO hospitals/health centres			(26.6–27.0)			(277.9–284.3)	
N	3,499	3,740		5,999	2,238		

*on-set of fever with shivering and remittance with sweating.

Table 2 shows that malaria-related knowledge was contingent on the level of household wealth (calculated on the basis of wealth quintiles). Thus, the top 20% households fared better than the bottom 20% with respect to most of the knowledge indicators. This was especially evident for knowledge on malaria transmission (three to four times increased in 2012 compared to 10–20% in 2007) and location of free treatment facilities (10–20% difference in 2012) in the follow-up survey. During this period, knowledge on the preventive usefulness of insecticidal bed nets increased greatly, more so among the bottom quintile.

**Table 2:** Knowledge on different aspects of malaria by wealth ranking %.

	High endemic districts				Low endemic districts			
	2007		2012		2007		2012	
Wealth Quintiles>	Bottom 20%	Top 20%	Bottom 20%	Top 20%	Bottom 20%	Top 20%	Bottom 20%	Top 20%
*Knowledge on malaria*								
Knows that malaria is caused by bite of mosquito	84.8	95.5	90.9	98.2	92.2	96.7	96.7	96.7
Knows that malaria is transmitted by the bite of mosquito which has bitten a malaria patient	30.5	43.0	10.2	41.7	23.7	43.7	11.6	33.3
Knows the most common symptom of Malaria*	68.7	82.9	90.7	97.6	76.3	86.5	89.0	94.4
Knows that insecticidal bed nets prevent malaria	0.7	1.2	84.6	78.6	0.9	2.3	84.6	78.6
Knows that malaria is treated by allopathic medicine	92.6	98.6	98.0	99.9	97.2	98.6	100.0	99.4
Knows that malaria treatment is available in public and NGO hospitals/health centres	—	—	47.8	62.4	—	—	48.1	58.8
N	884	651	853	717	1082	1294	337	486

*on-set of fever with shivering and remittance with sweating.

Over time, more and more people with malaria-like fever opted for qualified doctors in low endemic districts (from 13 to 28%) and paraprofessionals in both categories of districts (from 4 to 34% in high endemic and from 3 to 11% in low endemic districts) in preference to seeking treatment from unqualified drug store salespeople, seeking self-treatment or no treatment at all (Table 3).

**Table 3:** Key malaria practice indicators in 2007 (baseline) and 2012 (follow-up) by areas %.

	High endemic districts			Low endemic districts		
			χ^2^ value			χ^2^ value
	2007	2012	(95% CI)	2007	2012	(95% CI)
*Patients having malaria-like fever*						
Sought care from MBBS doctors	13.0	12.2	—	12.9	27.8	69.8
(for ex-malaria patients)						(69.3–70.4)
Paraprofessionals*	3.6	34.1	48.9	3.0	11.1	17.2
			(48.5–49.3)			(17.2–17.4)
Drug store salespeople	32.3	22.0	2.0	47.0	27.8	250.3
			(0.2–18.5)			(247.6–253.1)
Self-treatment	10.8	9.8	—	13.6	16.7	—
No treatment sought	38.7	22.0	4.2	22.7	5.6	123.3
			(1.3–13.4)			(122.1–124.5)
N	362	41		132	18	

*semi-qualified allopathic providers such as medical assistants and community health workers who have had some institutional training from government or NGOs.

Table 4 presents the maiden (the very first) source of information on malaria in two different periods. During the study period, the importance of neighbours/relatives/friends increased over time (around 40% to >80%) in both areas. In the high endemic districts, the importance of audiovisual media and CHWs decreased (from 15 to 8% and 48 to 38% respectively) during this period while the importance of print media increased, albeit marginally (from 3 to 5%).

**Table 4:** First source of information on malaria in 2007 (baseline) and 2012 (follow-up) by areas %.

	High endemic districts			Low endemic districts		
			χ^2^ value			χ^2^ value
	2007	2012	(95% CI)	2007	2012	(95% CI)
*First source of information*						
Print media (poster, leaflet, bill boards etc.)	2.7	4.9	25.7	2.0	1.9	—
			(25.6–25.9)			
						
Audiovisual media (radio, TV, cinema etc.)	15.0	8.0	89.8	15.2	20.4	31.8
			(89.1–90.7)			(31.6–32.1)
						
Community health workers	48.4	37.9	84.4	41.9	34.7	35.4
			(83.7–85.2)			(35.2–35.7)
						
Neighbours/relatives/friends	36.5	86.5	1978.5	49.3	80.0	630.9
			(1948.8–2008.8)			(622.9–639.1)
						
Others*	22.2	7.6	315.6	18.7	29.5	112.8
			(312.1–319.3)			(111.8–114.0)
						
N	3,750	3,745		5,999	2,250	

*include dissemination through loud-speakers in gatherings and some irregular instances in folk songs, popular theatre etc.

Finally, a logistic regression was run to explore factors predicting correct knowledge of the respondents on malaria (Table 5).This was found to be predicted by sex (more if male), household wealth status (improved if affluent), schooling (better if had schooling), and source of information. Interestingly, interpersonal communication between clients/respondents and the community health workers/neighbours/relatives/friends were found to be a significant predictor of correct knowledge on malaria compared to different mass media. Education was twice as important for predicting correct malaria knowledge, besides affluence (thrice as important in case of the least poor).

**Table 5:** Logistic regression of factors predicting correct knowledge on malaria* (n = 5,995).

	Exp (β)	95% confidence interval (CI)
Age in years (continuous)	0.99	0.98–1.00
Sex (‘0’ if female, ‘1’ if male)	1.00	1.30–1.95
Wealth Quintiles		
‘0’ if 1^st^ quintile (poorest)		
2^nd^ quintile	1.35	.89–2.05
3^rd^ quintile	1.40	.93–2.11
4^th^ quintile	2.48	1.68–3.66
5^th^ quintile (least poor)	3.45	2.34–5.02
Education (‘0’ if no education, ‘1’ if had education)	1.88	1.47–2.40
Source of information on malaria		
‘0’ if no information source		
Media (print and audiovisual)	1.27	0.99–1.64
Interpersonal communication (community health workers, Neighbours/relatives/friends)	2.19	1.25–3.85
Others (miking, dissemination at gathering of people, fair etc.)	1.10	0.68–1.81

*Correct knowledge if knows that i) malaria is caused by mosquito bite, ii) transmitted by the bite of a mosquito which has bitten a malaria patient, iii) prevented by insecticidal bed net, iv) treated by allopathic medicine and v) malaria diagnosis and treatment is available free of cost from public and NGO health facilities.

## Discussion

The follow-up survey shows improvement in some of the knowledge indicators such as the most common symptom suggestive of malaria, comparable to other countries such as eastern Sudan [16], Nepal [17], Turkey [18] and Iran [19]. Also, the dramatic improvement in knowledge regarding the importance of insecticidal bed nets for the prevention of malaria following awareness campaigns has also been observed in other studies [17], [20]. However, decline was observed for some critical indicators such as mode of malaria transmission and familiarity with the places where malaria treatment is available free of cost. This phenomenon of superficial knowledge without depth was also observed in another study on the use of insecticidal bed nets in high endemic upazilas from the same programme areas [21]. This should raise concern since this may interfere with taking informed actions for the treatment and prevention of malaria by the community [22], [23].

As the results showed, inequity in knowledge and practice was quite common depending upon economic condition of the households, location of the households in high or low endemic districts, and gender. The poorest quintile of the respondents lagged behind the top quintile in critical knowledge on malaria e.g., transmission, prevention by insecticidal nets, and familiarity with available free malaria care facilities. This was also true for possession and use of bed nets for vulnerable groups such as under-five children and pregnant women, in both areas (data not shown). This is not new, and has been discussed in literature [24], [25].

In treatment-seeking, the poorest quintile of the respondents was far behind in accessing professional/para-professional allopathic care compared to the top quintile of respondents especially in the high endemic districts (data not shown). However, more and more people with presumed malaria-like fever had opted for treatment from qualified doctors and para-professionals than other types of healthcare providers. This is a change in right direction since the lack of capacity of the non-qualified providers such as drug store salespeople to provide rational treatment is well documented [11].

Of the different media used (e.g., print, audio-visual, etc.), interpersonal communication with the community health workers, relatives, neighbours and friends was found to be increasingly dominant source of acquiring malaria information during the study period. In low endemic districts, dissemination of information through loud-speakers in mass gatherings such as weekly rural markets or fairs etc. were increasingly important source of information, but not in the high endemic districts. That the print and audiovisual media played little role in improving their understanding of malaria is shown by the low level of learning from these as reported by the respondents (data not shown). This is not surprising in a largely illiterate/semi-literate society, where the contents and forms of print and audio-visual materials may not be user-friendly or culture-sensitive, where dissemination through ‘words of mouth’ is more common, and interpersonal communication is more effective [6]. The main challenge lies in the lack of depth of malaria awareness and knowledge which is essential for taking informed action for prevention and treatment in the absence of programme inputs such as Long Lasting Insecticide-treated Nets (LLIN)/Insecticide Treated Nets(ITN), Rapid Diagnostic Tests (RDT) and drugs for malaria treatment] which are currently supplied free of cost. Thus, the programme need to revisit its IEC strategy to improve the depth of knowledge of the community and give attention where it is due i.e., interpersonal communication. In this regard, programme may also think of innovative means of information dissemination in this largely semi-literate community such as using folk songs, popular theatre, peer group discussion, etc.

### Limitations

This study focused on the communication channels used by the programme to explore its effect on improving malaria-related knowledge and practice. However, secular improvement in general health awareness from better socioeconomic condition and education, and relevant health education interventions from other agencies might have influenced the level of malarial knowledge and practice in the study population.

## Conclusion

The programme was successful in raising malaria awareness and knowledge in the community, albeit superficial, since the beginning of intervention in 2007. Conventional print and audiovisual media may not be user-friendly or culture-sensitive for this semi-literate/illiterate community where dissemination through ‘words of mouth’ is more common, and as such, interpersonal communication was found to be more effective. This is especially important for revisiting and redesigning communication strategy of the programme since in-depth knowledge is essential for taking informed decision. Besides, it has implications for sustainability of the programme in the future when free inputs won't be available.
